# Supplementary material for: Assessment of the functional state of the back muscles in girls with C-shaped low-grade scoliosis in a tensiomyographic image: An observational cross-sectional study
Source: PLoS One. 2023 Oct 17;18(10):e0292555. doi: 10.1371/journal.pone.0292555 (PMC10581459; doi:10.1371/journal.pone.0292555)
Supplement: S1 Protocol — (DOCX) [file pone.0292555.s002.docx]

**Assessment of the functional state of the back muscles in girls with C-shape low-grade scoliosis in a tensiomiographic image:** **an observational cross-sectional study**

1. **Project summary**

**Abstract**

**Objectives:** The aim of the study was to analyze the possible application of TMG in early detection of functional changes in back muscles in patients with low-grade scoliosis.

**Methods** Twenty five girls aged 13-15 took part in an observational (cross-sectional) study. The examination involved measurements using tensiomyography method (TMG). Two groups of muscles were tested: Latissimus Dorsi and Erector Spinae on the concave and convex side of low-grade scoliosis. The following indicators were analyzed: Td – delay time, Tc – contraction time and Dm – maximal muscle displacement.

**Results:** The analysis of Td revealed that values of this variable on the concave side were slightly lower compared to the convex side in both tested groups of muscles. Similar, Tc values on the concave side were slightly lower than on the convex side of the curvature in both studied muscles. In case of Dm, lower displacement values and, consequently, greater muscle rigidity were observed on the concave side of Latissimus Dorsi and the convex side of Erector Spinae. **Conclusions:** TMG method can be potentially used to diagnose the functional condition of muscles in patients with low-grade scoliosis. There were differences between the functional condition of the muscles on the concave and convex side of the curvature.

1. **General information**

**Assessment of the functional state of the back muscles in girls with C-shape low-grade scoliosis in a tensiomiographic image:** **an observational cross-sectional study**

Name and address of the sponsor/funder: None

Name and title of the investigator(s) who is (are) responsible for conducting the research, and the address and telephone number(s) of the research site(s), including responsibilities of each:

Katarzyna Ogrodzka-Ciechanowicz, Assoc. Prof.

**Institute of Clinical Rehabilitation,** Faculty of Motor Rehabilitation, University of Physical Education in Krakow

Al. Jana Pawla II 78,

31-571 Krakow, Poland

Mail: [katarzynaogrodzka@wp.pl](mailto:katarzynaogrodzka@wp.pl)

Name(s) and address(es) of the clinical laboratory(ies) and other medical and/or technical department(s) and/or institutions involved in the research:

Faculty of Motor Rehabilitation

The University of Physical Education

al. Jana Pawła II 78

31-571 Krakow, Poland

1. **Rationale & background information**

Scoliosis is a three-plane deformation of the spine and also the trunk, which may result in negative self-image, pain and potential negative effects connected with orthotic or surgical treatment [1].

There are several identified causes of scolioses, for example congenital or neuromuscular scolioses, but the most common one is adolescent idiopathic scoliosis (AIS) the cause of which has not been fully recognized. There are studies describing the role of genetics and biomechanics in developing scoliosis but the real cause of this disease remains largely unknown [2,3].

The onset and the development of scoliosis may depend on etiological and biomechanical factors [4]. Etiological factors may be very diverse and initiate scoliosis (IS). Biomechanical factor is, in turn, typical for all types of scolioses and acts according to the laws of gravity and growth regardless of etiology. This factor controls the development of the deformation [5].

However, some believe that original disorders connected with the development of scoliosis can be found in the central nervous system (CNS), what leads to muscle imbalance.

Thus, changes in the passive elements of the spine are secondary [6]. In scoliosis, the muscle balance of the main spine stabilizers, or spinal erectors, may be distorted due to original dysfunctions of the CNS structures [7]. This, in turn, may lead to disorders of the erector spinae muscles functioning and tension, causing curvatures of the spine. There are many arguments showing that discrete functional changes in the CNS are the actual cause of idiopathic scoliosis [8].

In both diagnostics and prevention of scoliosis, there is a strong focus on the assessment of differences in tension of the erector spinae muscles on the concave and convex side of scoliosis [9].

Determination of asymmetries (differences) in the erector spinae tensions between the concave and the convex side of the curvature seems to be essential when it comes to selecting the best conservative treatment method [10].

The muscular system is considered to be a coherent structure of co-dependent elements. There are many theories which explain the complex relations between the certain muscle groups and individual muscles. The majority of the contemporary theses is based on certain functional muscle and tendon chains [11].

Tensiomyography (TMG) is a method which enables precise measurement of contraction speed and muscle rigidity and assessment of the changes in muscle belly displacement during an electrically stimulated isometric contraction response.

This method of examination of muscle morphophysiology has been known for almost 20 years and allows to obtain information about the functioning of the muscle easier than from electromyography. It was developed in 1983 by Professor Vojko Valenčič.
Unlike EMG, it does not provide information about the activity of certain muscles in the studied function but as a noninvasive method, it allows detection of skeletal muscles’ properties. During the TMG, the changes in muscle belly displacement in millimeters (mm) and the duration expressed in milliseconds (ms) in response to a single electric stimulus are assessed [12,13,14].

TMG does not require any effort from the patient and therefore it is often used to asses the functions of the muscles after physical activity [15].

In the recent years, tensiomyography has become an important and highly reliable technique of assessing muscle contractile properties [16,17,18].

TMG is more and more often used in sports medicine, especially to assess the neuromuscular properties of various muscles as well as functional efficiency and recovery after effort [19,20,21].

However, there are no reports on using TMG in the assessment of spinal muscle contractile property, in particular in scolioses.

1. **References (of literature cited in preceding sections)**
2. Cheng, J.C., Castelein, R.M., Chu, W.C., Danielsson, A.J., Dobbs, M.B., Grivas, T.B., et al. Adolescent idiopathic scoliosis. *Nat Rev Dis Primers*. **24**(1), 15030 (2015).
3. Schlösser, T.P., van der Heijden, G.J., Versteeg, A.L & Castelein, R.M. How 'idiopathic' is adolescent idiopathic scoliosis? A systematic review on associated abnormalities. *PLoS One* **9**(5), e97461 (2014).
4. Gorman, K.F., Julien, C. & Moreau, A. The genetic epidemiology of idiopathic scoliosis. *Eur Spine J*. **21**(10), 1905-1919 (2012).
5. Schmid, S., Burkhart, K.A., Allaire, B.T., Grindle, D., Bassani, T., Galbusera, F., et. al. Spinal Compressive Forces in Adolescent Idiopathic Scoliosis With and Without Carrying Loads: A Musculoskeletal Modeling Study. *Front Bioeng Biotechnol.* **8**, 159 (2020).
6. Schreiber, S., Parent, E.C., Hill, D.L., Hedden, D.M., Moreau, M.J. & Southon, S.C. Patients with adolescent idiopathic scoliosis perceive positive improvements regardless of change in the Cobb angle – Results from a randomized controlled trial comparing a 6-month Schroth intervention added to standard care and standard care alone. SOSORT 2018 Award winner. *BMC Musculoskelet Disord.* **20**, 1–10 (2019).
7. Tylman, D. *Pathomechanics of Lateral Spinal Curvatures*. (ed. Severus, Poland) (1995).
8. Guo, L.Y., Wang, Y.L., Huang, Y.H., Yang, C.H., Hou, Y.Y., Harn, H.I., et al. Comparison of the electromyographic activation level and unilateral selectivity of erector spinae during different selected movements. *Int J Rehabil Res*. **35**, 345–351 (2012).
9. Perret, C. & Robert, J. Electromyographic Responses of Paraspinal Muscles to Postural Disturbance with Special Reference to Scoliotic Children. *J Manip Physiol Ther*. **27**, 375–380 (2004).
10. Weiss, H.R. Imbalance of electromyographic activity and physical rehabilitation of patients with idiopathic scoliosis. *Eur Spine J.* **1**, 240–243 (1993).
11. Kaplan, P.E., Sahgal, V., Hughes, R., Kane, W. & Flanagan, N. Neuropathy in Thoracic Scoliosis. *Acta Orthop Scand*. **51**, 263–266 (1980).
12. Richter P., Hebgen E. & Gieremek, K. *Trigger points and myofascial chains in osteopathy and manual therapy*. (ed. Galaktyka, ed. 2) (2014).
13. Pakosz, P., Jakubowska-Lukanova, A. & Gnoiński, M. TMG As a Prevention Method of Athletes Muscles, Ligaments and Joints Injuries. *Pol J Sports Med*. **32**, 189–200 (2016).
14. Pakosz, P., Konieczny, M. & Gnoiński, M. *Change in the neuromuscular profile of the lower limbs after a 15-minute warm-up, assessed by TMG, in young females group*. In: A Man In Health And Disease Health Promotion, (Care And Rehabilitation) 376-384 (2018).
15. Fryc, D. & Bibrowicz, K. Tensomiographic characteristics of selected muscles stabilizing the pelvis in correlation with the pelvis angle in young, healthy women. *Curr Prob Biomech*. **20**, 37-43 (2020).
16. Martín-San Agustín, R., Medina-Mirapeix, F., Casaña-Granell, J., García-Vidal, J.A., Lillo-Navarro, C., et al. Tensiomyographical responsiveness to peripheral fatigue in quadriceps femoris. *Peer J*. **8**, e8674 (2020).
17. Lohr, C., Schmidt, T., Medina-Porqueres, I., Braumann, K.M., Reer, R. & Porthun, J. Diagnostic accuracy, validity, and reliability of Tensiomyography to assess muscle function and exercise-induced fatigue in healthy participants. A systematic review with meta-analysis. *J Electromyogr Kinesiol.* **47**, 65–87 (2019).
18. Martín-Rodríguez, S., Loturco, I., Hunter, A.M., Rodríguez-Ruiz, D. & Munguia-Izquierdo, D. Reliability and Measurement Error of Tensiomyography to Assess Mechanical Muscle Function: A Systematic Review. *J Strength Cond Res*. **31**, 3524-3536 (2017).
19. Valencic, V. & Knez, N. Measuring of skeletal muscles’ dynamic properties. *Artif Organs* **21**(3), 240–242 (1997).
20. Alvarez-Diaz, P., Alentorn-Geli, E., Ramon, S., Marin, M., Steinbacher, G., Rius M, et al. Comparison of tensiomyographic neuromuscular characteristics between muscles of the dominant and non-dominant lower extremity in male soccer players. *Knee Surg Sports Traumatol Arthrosc*. **24**, 2259-2263 (2016).
21. Pereira, L.A., Ramirez-Campillo, R., Martín-Rodríguez, S., Kobal, R., Abad, C.C., Arruda A.F.S, et al. Is tensiomyography-derived velocity of contraction a sensitive marker to detect acute performance changes in elite team-sport athletes? *Int J Sports Physiol Perform*. **15**(1), 31–37 (2020).
22. García-Sillero, M., Benítez-Porres, J., García-Romero, J., Bonilla, D.A, Petro J.L.& Vargas-Molina, S. Comparison of interventional strategies to improve recovery after eccentric exercise-induced muscle fatigue. *Int J Environ Res Public Health*. **18**(2), 1–11 (2021).
23. **Study goals and objectives**

There are no reports on using TMG in the assessment of spinal muscle contractile property, in particular in scolioses. That is why the aim of the study was to analyze the possible application of TMG in early detection of functional changes in back muscles in patients with low-grade scoliosis.

1. **Study design**

This is an observational (cross-sectional) study. The study protocol follows the guidelines of the Helsinki Declaration. This study was conducted in compliance with the Strengthening the Reporting of Observational Studies in Epidemiology (STROBE) Statement: guidelines for reporting observational studies.

The first author had qualified 30 female patients of a physiotherapy clinic, who were beginning their low-grade scoliosis therapy. The study was conducted in July 2021, during a rehabilitation holiday in Sarbinowo, organized by Orto-Med clinic from Bielsko-Biała. Children with low-grade lumbar scoliosis diagnosed by an orthopedist according to SOSORT guidelines participated in the preventive and therapeutic stay.


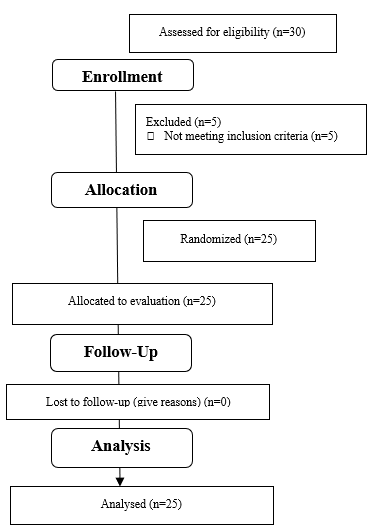


Eligibility criteria:

- Gender: female;
- Low-grade lumbar adolescent idiopathic scoliosis diagnosed by an orthopedist, the mean curvature angle between 11°-15° according to Cobb (Mean = 13.2° SD = 1.4); Risser ≤ 3 (based on the subject examination and X-ray examination with determination of the Cobb angle);
- Patients have had their first menstruation;
- No coexisting diseases which could affect the test results;
- Written consent of a parent (guardian) for the patient to participate in the study.

All the participants declared that their physical-recreational activity is moderate and that they were not engaged in competitive sport.

1. **Methodology**

The examination involved measurements using tensiomyography method. Two groups of muscles were tested: Latissimus Dorsi (Lat. Dorsi) and Erector Spinae (Er. Spinae) on the concave and convex side of low-grade scoliosis.

The following indicators were analyzed:

Td – delay time, determined between the electric impulse and 10% of the muscle response (ms)

Tc – contraction time, from 10% to 90% of the maximal response from the muscle, measured in milliseconds (ms)

Dm – maximal muscle displacement, measured in millimeters (mm).

Two self-adhesive electrodes, 2-4 cm in diameter, were attached on the subjects’ bodies. Electrode diameter was selected based on the muscle size and to isolate contraction of a specific muscle and avoid activation of the muscles located nearby. A single one-phase square impulse of 1 ms duration was delivered from the electrostimulator to the electrode to evoke the muscle contraction transdermally. The impulse power was gradually increased by 10 mA until a peak muscle contraction response was obtained. Typical maximal responses were observed between 40 and 90 mA. In order to minimize fatigue effects, 10-second intervals were maintained between each stimulation impulse. The area where the sensor was attached was chosen according to TMG scheme, in the thickest part of the muscle. If necessary, the area of sensor attachment was slightly corrected afterwards to obtain the highest mechanical response. The sensor adhered to the skin half distance between the electrodes, about 5 cm from the center of the electrodes.

The digital TMG signal was generated directly from the sensor using sapling frequency of 1 kHz. After the measurement, the TMG signal was stored on a PC drive. Maximum values obtained from two measurements were recorded and averaged for further analyses. The accepted maximal stimulation amplitude was the minimal amplitude needed for the response with the greatest muscle displacement (Dm).

The tests were performed in a physician’s office between 8 a.m. and 13 p.m. The examined person assumed the prone position with their arms along the body, the head in a position enabling relaxation of the whole body and with rollers placed under the talocrural joints. The electrodes were attached along the muscle bellies of latissimus dorsi and erector spinae on both sides of the curvature. The contraction time was evoked by single electric stimuli. Self-adhesive electrodes were placed around the TMG sensor. The anode was attached distally and the cathode – proximally, 20-50 mm from the measurement point. Bipolar electric stimulation involved a single direct current impulse which lasted 1 millisecond.

1. **Safety considerations**

The tests were carried out with the written consent of the patient and in accordance with the guidelines of the Bioethics Committee. Individual test results were known only to the research manager.

1. **Follow-up**

Research includes the analysis of the possible application of TMG in early detection of functional changes in back muscles in patients with low-grade scoliosis. After analysis, no long-term changes that would affect the patient are observed.

1. **Data management and statistical analysis**

The statistical analysis of the results was conducted using MedCalc ver. 20.015 package. The analysis of the results and their description was carried out by the authors of the publication in the conditions of a blinded trial. The sample size was not specified, the study was conducted on all participants of the rehabilitation stay who met the criteria for inclusion in the study. The characteristics of the distribution of variables were determined by means of Shapiro-Wilk method. Mean values and standard deviation were calculated. As the distribution of the variables was normal, the analysis of the bilateral variables was conducted using Student’s t-test for independent samples. Differences were considered significant at p < 0.05.

1. **Quality assurance**

The research was approved by by the Bioethics Committee at the Kazimiera Malinowska College of Education and Therapy in Poznań, No. 003/2019 (approval date 15.02.2019). The studies did not concern drug testing, nor did it affect the patient's skin continuity. The subjects were under medical supervision.

1. **Expected outcomes of the study**

The results of the article indicate the TMG method can be potentially used to diagnose the functional condition of muscles in patients with low-grade scoliosis. There were differences between the functional condition of the muscles on the concave and convex side of the curvature. The study did not show clear differences in the functional condition of Erectus Spinae and Latissimus Dorsi among the examined patients.

**Dissemination of results and publication policy**

Publication of research results in a scientific journal will allow their dissemination. All the authors of the article are academic employees of universities, which will allow to disseminate research results among students and other researchers (lectures for students of physiotherapy and occupational therapy, lectures at conferences and scientific meetings), or practicing physiotherapists, which will allow the dissemination of research results among other members interdisciplinary team.

1. **Duration of the project**

The research was completed before completing the WHO protocol, lasted one month

1. **Problems anticipated**

The research was completed before completing the WHO protocol. The researchers did not encounter any financial problems.

**Project management**

Tomasz Szurmik: research idea, research plan development, patient recruitment, research, data collection, data analysis, literature search, manuscript writing, manuscript approval.

Katarzyna Ogrodzka-Ciechanowicz: searching literature, manuscript writing, manuscript approval.

Piotr Kurzeja: data analysis, manuscript writing, manuscript approval.

Bartłomiej Gąsienica-Walczak: searching literature, manuscript approval.

Jarosław Prusak: searching literature, manuscript approval.

Karol Bibrowicz: research idea, research plan development, research, data collection, data analysis, literature search, manuscript writing, manuscript approval.

1. **Ethics**

The study was approved by the Bioethics Committee at the Kazimiera Malinowska College of Education and Therapy in Poznań, No. 003/2019 (approval date 15.02.2019). In addition, each patient received a brief written description of the purpose and methodology of the study. Each patient was informed in detail about their rights, had the opportunity to ask questions throughout the entire duration of the project and had the opportunity to resign from participation in the project at any time of their choice. Each patient/ legal guardian signed a consent to voluntary participation in the project.

1. **Informed consent forms**

**Intervention**

Two self-adhesive electrodes, 2-4 cm in diameter, were attached on the subjects’ bodies. Electrode diameter was selected based on the muscle size and to isolate contraction of a specific muscle and avoid activation of the muscles located nearby. A single one-phase square impulse of 1 ms duration was delivered from the electrostimulator to the electrode to evoke the muscle contraction transdermally. The impulse power was gradually increased by 10 mA until a peak muscle contraction response was obtained. Typical maximal responses were observed between 40 and 90 mA. In order to minimize fatigue effects, 10-second intervals were maintained between each stimulation impulse. The area where the sensor was attached was chosen according to TMG scheme, in the thickest part of the muscle. If necessary, the area of sensor attachment was slightly corrected afterwards to obtain the highest mechanical response. The sensor adhered to the skin half distance between the electrodes, about 5 cm from the center of the electrodes.

The digital TMG signal was generated directly from the sensor using sapling frequency of 1 kHz. After the measurement, the TMG signal was stored on a PC drive. Maximum values obtained from two measurements were recorded and averaged for further analyses. The accepted maximal stimulation amplitude was the minimal amplitude needed for the response with the greatest muscle displacement (Dm).

The tests were performed in a physician’s office between 8 a.m. and 13 p.m. The examined person assumed the prone position with their arms along the body, the head in a position enabling relaxation of the whole body and with rollers placed under the talocrural joints. The electrodes were attached along the muscle bellies of latissimus dorsi and erector spinae on both sides of the curvature. The contraction time was evoked by single electric stimuli. Self-adhesive electrodes were placed around the TMG sensor. The anode was attached distally and the cathode – proximally, 20-50 mm from the measurement point. Bipolar electric stimulation involved a single direct current impulse which lasted 1 millisecond.

1. **Budget**

This research did not receive any specific grant from funding agencies in the public, commercial, or not-for-profit sectors.

1. **Other support for the project**

Not applicable

1. **Collaboration with other scientists or research institutions**

Not applicable

1. **Links to other projects**

Not applicable

1. **Curriculum Vitae of investigators**

**Tomasz Szurmik, PhD**

Mail: [info@orto-med.com.pl](mailto:info@orto-med.com.pl)

**Education:**

Master's degree in Physiotherapy, Podhale State College of Applied Sciences in Nowy Targ, Institute of Health Sciences

**Professional work:**

Faculty of Arts and Educational Science, University of Silesia, Cieszyn,

Orto-med – Private physiotherapy practice

**Katarzyna Ogrodzka-Ciechanowicz, Assoc. Prof.**

Mail: [katarzyna.ogrodzka@awf.krakow.pl](mailto:katarzyna.ogrodzka@awf.krakow.pl)

**Education:**

Master's degree in Physiotherapy, the University of Physical Education

Master's degree in Physical Education, the University of Physical Education

**Professional work:**

University of Physical Education in Krakow, Department of Movement Rehabilitation, Section of Rehabilitation in Traumatology

Podhale State College of Applied Sciences in Nowy Targ, Institute of Health Sciences

Cracow University of Health Promotion, Physiotherapy studies, Podology course

Jagiellonian University Medical College, Faculty of Medicine

College of Administration in Bielsko-Biała, Department of Physiotherapy

Physiotherapist at Krakow Center for the Diagnosis of the Spine

**Piotr Kurzeja, PhD**

Mail: [piotrkurzeja@op.pl](mailto:piotrkurzeja@op.pl)

**Education:**

Medical School Faculty of Physiotherapy

Master's degree in Physiotherapy, University of Physical Education in Krakow

**Professional work:**

Podhale State College of Applied Sciences in Nowy Targ, Institute of Health Sciences

Private physiotherapy practice

Silesian Rehabilitation Center in Rabka-Zdrój

**Bartłomiej Gąsienica-Walczak, PhD**

Mail: [bgw01@interia.pl](mailto:bgw01@interia.pl)

**Education:**

Master of Physiotherapy, University of Physical Education in Krakow.

**Professional work:**

Podhale State College of Applied Sciences in Nowy Targ, Institute of Health Sciences

**Jarosław Prusak, PhD**

Mail: [j.prusak2020@gmail.com](mailto:j.prusak2020@gmail.com)

**Education**:

Master's degree in physical education, specialization in biological regeneration, Academy of Physical Education. Jędrzej Śniadecki in Gdańsk.

Bachelor's degree in physiotherapy, Cracow University of Health Promotion.

**Professional work:**

Institute of Tuberculosis and Lung Diseases OT in Rabka-Zdrój,

Podhale State College of Applied Sciences in Nowy Targ, Institute of Health Sciences

**Karol Bibrowicz, PhD**

Mail: [bibrowicz@wp.pl](mailto:bibrowicz@wp.pl)

**Education:**

Master of Physiotherapy, University of Physical Education in Wrocław

**Professional work:**

Science and Research Center of Body Posture, Kazimiera Milanowska College of Education and Therapy, Poznań

1. **Other research activities of the investigators**

At the moment, the principal researcher is not participating in any project.

1. **Financing and insurance**

Not applicable
